# Disparities in access to primary care are growing wider in Canada

**DOI:** 10.1177/08404704231183599

**Published:** 2023-06-20

**Authors:** M. Ruth Lavergne, Aidan Bodner, Sara Allin, Erin Christian, Mohammad Hajizadeh, Lindsay Hedden, Alan Katz, George Kephart, Myles Leslie, David Rudoler, Sarah Spencer

**Affiliations:** 1 3688 Dalhousie University, Halifax, Nova Scotia, Canada.; 2 7938 University of Toronto, Toronto, Ontario, Canada.; 3 3682 IWK Health Centre, Halifax, Nova Scotia, Canada.; 4 1763 Simon Fraser University, Burnaby, British Columbia, Canada.; 5 8664 University of Manitoba, Winnipeg, Manitoba, Canada.; 6 2129 University of Calgary, Calgary, Alberta, Canada.; 7 85458 Ontario Tech University, Oshawa, Ontario, Canada.

## Abstract

Canadian provinces and territories have undertaken varied reforms to how primary care is funded, organized, and delivered, but equity impacts of reforms are unclear. We explore disparities in access to primary care by income, educational attainment, dwelling ownership, immigration, racialization, place of residence (metropolitan/non-metropolitan), and sex/gender, and how these have changed over time, using data from the Canadian Community Health Survey (2007/08 and 2015/16 or 2017/18). We observe disparities by income, educational attainment, dwelling ownership, recent immigration, immigration (regular place of care), racialization (regular place of care), and sex/gender. Disparities are persistent over time or increasing in the case of income and racialization (regular medical provider and consulted with a medical professional). Primary care policy decisions that do not explicitly consider existing inequities may continue to entrench them. Careful study of equity impacts of ongoing policy reforms is needed.

## Introduction

Strong primary care systems have been associated with greater equity in population health, while contributing to better patient outcomes, provider experiences, and efficiency of health systems,^1-6^ but inequities in access to primary care may be growing. Primary care is the first and main point of access to healthcare in Canada and so more equitable access to primary care (here defined as similar access to services for people with similar needs regardless of social position^2,3,7,8^) has the potential to improve equity in access throughout the system, and ideally contribute to more equitable health outcomes.^1^ We note “primary care” is distinct from “primary healthcare” which has a broader population orientation and equity as a core principle.^9-11^ We use the term “primary care” as it more accurately describes services in Canada.

Canadian provinces and territories have undertaken varied macro- and meso-level reforms to how primary care is organized, financed, delivered, and held accountable.^1,12-15^ Ideally these efforts would promote more equitable access to primary care. However, in implementing new models of care, areas with resources and readiness for change may be prioritized for feasibility, even if they are not areas with greatest need.^14^ It may also be that people in positions of advantage can more easily navigate access to innovative models.^1^ Indeed, research shows that people experiencing economic or social marginalization were less likely to be enrolled in new models in Ontario^16,17^ and Alberta.^18^ In British Columbia, primary care visits, continuity, and specialist referrals fell more rapidly in low-income neighbourhoods relative to high-income neighbourhoods over a period of reform.^19^ Similarly, in Ontario, cancer screening gaps by income quintile grew wider in the context of primary care payment reform.^20^ National studies have highlighted inequities in healthcare service use^21-23^ and persistent reports of unmet need^24^ in Canada.

This study builds on this research, contributing national data documenting the extent to which access to primary care by income, educational attainment, dwelling ownership, immigration, racialization, sex/gender, and urban/rural residence has changed in Canada over a time of ongoing primary care reform.

## Methods

### Study data and population

We used data from the Canadian Community Health Survey (CCHS), a national health survey of people residing in all provinces and territories in Canada. We accessed the Public Use Microdata File (PUMF), a publicly available version of the CCHS file^25-27^ and so ethics approval for this study was not required. We use data from 2007/08 and 2015/16 or 2017/18 (depending on the measure of access, described below). These years were selected as the earliest and latest for which comparable variables describing access to primary care and measures of social position were available. This also captures a period of sustained primary care reform, but does not include changes in the context of the COVID-19 pandemic. All survey respondents in the selected years were included in the study.^28-30^ The CCHS does not include people younger than 12 years of age, Indigenous people living on reserve, people who are institutionalized, youth living in foster homes, members of the armed forces, and people who live in some remote regions.^25-27^

#### Measuring access to primary care

We identified items that inform the process of accessing primary care, and that can be compared over time. Chosen questions are informed by the framework proposed by Levesque et al.,^31^ which identifies stages in the process of accessing care. The questions “Do you have a regular medical provider?” and “Do you have a regular place of care?” focus on availability of primary care services. We include having consulted with a medical professional as a measure of use of health services, though this does not specify if this was with a primary care provider or if the consultation was episodic in nature or part of a continuous relationship. This was asked in the 2015/16 CCHS but not the 2017/18 CCHS.

#### Stratification variables

Stratification variables were informed by the Canadian Institute for Health Information (CIHI) list of equity stratifiers, reflecting measures of social position relevant to health and healthcare use.^32^ We identified variables that were consistently measured across survey years. We dichotomized variables to support clarity and interpretation, given the number of variables examined. Categories are as follows: household income (low vs. middle/high), household education level (secondary school graduation or less vs. post-secondary), household dwelling ownership (dwelling owned by household member vs. dwelling not owned by household member), immigration (recent or non-recent immigrant vs. born in Canada), racialization (White or racialized), and location of residence, based on health region (metropolitan and non-metropolitan).

There are important limitations of these variables we wish to emphasize. Respondents were asked if they are male or female. It is not clear whether this would be interpreted by respondents as legal sex, sex assigned at birth, or gender, so we label this sex/gender to reflect this uncertainty. Only a binary variable reflecting racialization is available, with all people not racialized as White grouped together. While we label these categories “White” and “racialized,” we recognize the potential harm in framing Whiteness as a default, and also that this labelling does not reflect that Whiteness itself is racialized in ways that confer power and privilege in the Canadian context. Location of residence was assigned based on health region of residence. Health regions that include a Census Metropolitan Area^33^ were classified as metropolitan, all others were non-metropolitan. This reflects residence in health regions that likely have more extensive resources for healthcare, including hospitals and tertiary care centres, but does not reflect individual rural/urban residence as the units of health regions are large and may include both urban and rural places.

#### Measuring health need

Equity is often defined as equal access for equal need. In multivariable analysis, we include variables that may be associated with stratifiers and also with need for primary care,^17,34^ including age, sex/gender (female/male), self-reported health (excellent, good, fair, or poor), and presence of chronic conditions (a series of binary variables reflecting having been told by a medical professional respondents have asthma, arthritis, high blood pressure, diabetes, heart disease, cancer, stroke effects, anxiety disorders, and/or mood disorders).

### Statistical analysis

We calculated the percent of people with access for each of the three measures, stratified by measures of social position at both the earlier and later time periods, applying survey weights in all descriptive analysis. Respondents with missing responses for service use variables were not included in analysis. We calculate the difference in percentages over time and by stratifier. We report the difference in differences over time, with negative values reflecting growing disparities. We also graph change over time to show patterns visually. A note that we use “disparities” to describe differences in primary care access or use within reporting of quantitative methods and results. We use “inequities” to reflect our interpretation that these differences are unfair and unjust, as they reflect differences by social position and not need for healthcare.

For each access measure and measure of social position, we constructed a binary logistic regression model examining odds of access (greater access: 1 and less access: 0), including data from both time periods. Each model included a binary variable for year (most recent vs. earliest), the measure of social position (with the reference being the group with relative advantage), an interaction between the year and measure of social position, and measures of need (sex/gender, self-reported health, and chronic conditions). The estimated odds ratios for each measure of social position informed whether we observe a disparity in access overall (OR < 1 comparing groups that experience marginalization to those with relative advantage), whether access declined over time generally (OR < 1), and whether declines were greater for groups with lower social position (OR < 1) (i.e. if disparities grew wider over time). We applied scaled survey weights in multivariable analysis,^27^ using the “survey” package^35^ in R version 4.1.1 with standard errors corrected for cluster sampling.^36,37^

We performed two additional analyses to confirm consistency of findings. We removed sex/gender as an adjustment variable and stratified by sex/gender to determine if changes over time differ by sex/gender categories. We also completed the main analysis stratified by province/territory to confirm findings are similar across settings.

## Results

**Figure 1.**
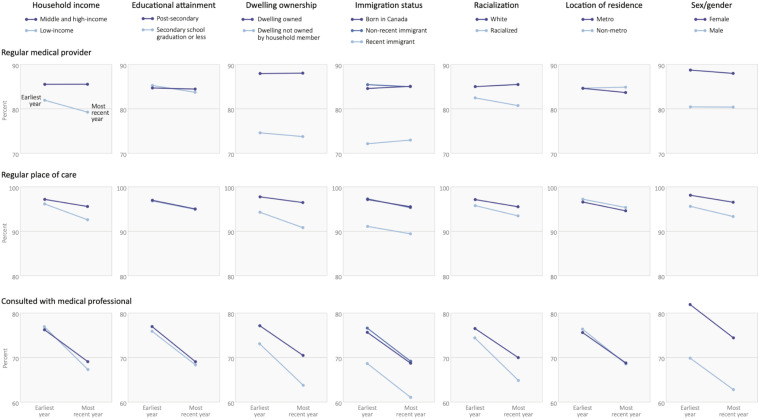
Changes in primary care access by measure of social position.

In 2007/8, the percentage of people with a regular medical provider was lower among people with low income (82.01% vs. 85.53%), who live in dwellings not owned by a household member (74.62% vs. 87.96%), who were recent immigrants (72.11% vs. 84.57% born in Canada), who were racialized as non-White (82.51% vs. 85.00%), and among respondents described as male (80.42% vs. 88.76%), (Table 1, Figure 1). The percentage of people with a regular medical provider or place of care differed less by educational attainment and metro/non-metro residence (differences of .61 and -.01 percentage points, respectively). Similar patterns were observed when examining the percentage of participants with a regular place of care. In 2007/8, a slightly higher percentage of people with low income had consulted a medical professional (77.18% vs. 76.45%), but patterns were otherwise similar to other access variables. Over time, disparities based on income, dwelling ownership, and racialization grew visibly wider (Figure 1).

**Table 1.** Differences in access to primary care in Canada by social position and over time, 2007/8 to 2017/8 (regular medical provider and place of care) or 2015/6 (consulted with medical professional).

	Regular medical provider			Regular place of care			Consulted with medical professional		
	2007/8	2017/18	% change	2007/8	2017/18	% change	2007/8	2015/16	% change
Household income									
Low-income	82.01	79.15	−2.86	96.26	92.47	−3.79	77.18	67.07	−10.11
Middle- and high-income	85.53	85.55	.03	97.23	95.52	−1.71	76.45	68.88	−7.57
Difference (low-income vs. middle- and high-income)	−3.52	−6.40	−2.89	−.97	−3.05	−2.08	.73	−1.81	−2.55
Educational attainment									
Post-secondary	84.72	84.45	−.27	97.04	94.96	−2.08	77.18	68.85	−8.33
Secondary school graduation or less	85.33	83.69	−1.64	96.88	94.85	−2.03	76.11	68.15	−7.96
Difference (secondary school graduation or less vs. post-secondary)	.61	−.75	−1.37	−.15	−.11	.05	−1.07	−.70	.37
Dwelling ownership									
Dwelling not owned by household member	74.62	73.72	−.90	94.40	90.71	−3.70	73.33	63.54	−9.79
Dwelling owned by household member	87.96	88.05	.09	97.79	96.42	−1.37	77.35	70.29	−7.06
Difference (not owned vs. owned)	−13.34	−14.33	−.99	−3.39	−5.71	−2.33	−4.02	−6.75	−2.73
Immigration									
Non-recent immigrant	85.47	84.99	−.48	97.35	95.27	−2.07	76.83	68.96	−7.87
Recent immigrant	72.11	72.99	.87	91.16	89.38	−1.78	68.87	60.84	−8.03
Born in Canada	84.57	85.10	.53	97.20	95.46	−1.74	75.87	68.53	−7.35
Difference (recent immigrant vs. born in Canada)	−12.46	−12.11	.35	−6.04	−6.08	−.03	−7.00	−7.68	−.68
Racialization									
Racialized	82.51	80.65	−1.86	95.86	93.39	−2.47	74.66	64.61	−10.05
White	85.00	85.51	.51	97.19	95.46	−1.73	76.72	69.80	−6.92
Difference (racialized vs. White)	−2.49	−4.87	−2.37	−1.33	−2.08	−.74	−2.06	−5.19	−3.13
Sex/Gender									
Female	88.76	87.96	−.80	98.16	96.48	−1.68	82.13	74.22	−7.91
Male	80.42	80.38	−.05	95.69	93.24	−2.44	70.07	62.59	−7.48
Difference (male vs. female)	−8.34	−7.58	.76	−2.47	−3.23	−.76	−12.06	−11.63	.43
Health region									
Metro	84.66	83.65	−1.01	96.65	94.54	−2.11	75.82	68.58	−7.24
Non-metro	84.65	84.90	.25	97.28	95.29	−1.99	76.61	68.35	−8.25
Difference (non-metro vs. metro)	−.01	1.25	1.26	.63	.75	.12	.79	−.23	−1.02

**Figure 2.**
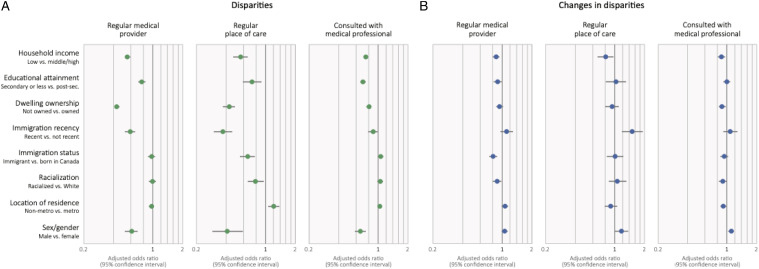
Adjusted odds ratios reflecting changes disparities in access, and changes in disparities in Canada, 2007/8 to 2017/8 (regular medical provider and place of care) or 2015/6 (consulted with medical professional). Note: odds ratios below 1 in panel (a) reflect disparities in access to care, and odds ratios below 1 in panel (b) reflect growing disparities in access. All models adjusted for age, sex/gender, self-reported health (excellent, good, fair, or poor), and chronic conditions (asthma, arthritis, high blood pressure, diabetes, heart disease, cancer, stroke effects, anxiety disorders, and/or mood disorder.

Multivariable models adjusting for variables reflecting need for care reinforce observations of disparities by income, educational attainment, dwelling ownership, recent immigration, immigration (regular place of care), racialization (regular place of care), and sex/gender (Figure 2, Table 2). Interaction terms show disparities persisted over time or increased in the case of income and racialization (regular medical provider, consulted with a medical professional). Exceptions are that disparities in access based on recent immigration attenuated, as did differences by sex/gender. People in non-metropolitan health regions had higher odds of having a regular place of care overall (OR 1.21; 1.06, 1.38), though we did not observe differences for other access variables.

**Table 2.** Adjusted odds ratios reflecting changes in access over time, disparities in access, and changes in disparities in Canada, 2007/8 to 2017/8 (regular medical provider and place of care) or 2015/6 (consulted with medical professional).

		Regular medical provider			Regular place of care			Consulted with medical professional		
			95% CI			95% CI			95% CI	
		OR	Lower	Upper	OR	Lower	Upper	OR	Lower	Upper
Household income										
Low-income (vs. middle and high-income)	Change over time	.90	.86	.94	.55	.50	.61	.60	.57	.62
	Disparity	.55	.52	.59	.56	.47	.66	.75	.71	.79
	Change in disparity	.87	.80	.94	.81	.67	.97	.88	.81	.96
Educational attainment										
Secondary school graduation or less (vs. post-secondary)	Change over time	.93	.89	.97	.55	.50	.60	.60	.58	.62
	Disparity	.77	.71	.84	.73	.59	.91	.70	.66	.75
	Change in disparity	.90	.82	.99	1.03	.81	1.30	1.00	.92	1.08
Dwelling ownership										
Dwelling not owned by household member (vs. dwelling owned by household member)	Change over time	.95	.90	.99	.57	.52	.64	.62	.60	.65
	Disparity	.43	.41	.46	.43	.37	.49	.81	.76	.85
	Change in disparity	.94	.87	1.02	.94	.80	1.10	.89	.83	.97
Recent immigration										
Recent immigrant (vs. non-recent immigrant)	Change over time	.90	.87	.94	.52	.48	.56	.60	.58	.62
	Disparity	.59	.52	.66	.37	.30	.46	.89	.79	.99
	Change in disparity	1.11	.96	1.29	1.50	1.18	1.92	1.08	.92	1.28
Immigration										
Immigrant (vs. born in Canada)	Change over time	.97	.93	1.01	.56	.52	.61	.61	.59	.63
	Disparity	.97	.89	1.05	.66	.55	.78	1.06	.99	1.13
	Change in disparity	.81	.74	.89	1.01	.83	1.22	.94	.86	1.03
Racialization										
Racialized (vs. White)	Change over time	.95	.92	.99	.55	.51	.60	.62	.59	.64
	Disparity	.99	.91	1.07	.79	.66	.96	1.05	.98	1.12
	Change in disparity	.89	.80	.98	1.06	.87	1.31	.91	.83	1.00
Sex/Gender										
Male (vs. female)	Change over time	.88	.83	.92	.49	.44	.56	.58	.55	.60
	Disparity	.61	.52	.70	.41	.29	.59	.66	.58	.75
	Change in disparity	1.06	.99	1.14	1.17	1.00	1.37	1.11	1.03	1.18
Metropolitan/Non-metropolitan health region										
Non-metro (vs. metro)	Change over time	.88	.83	.93	.57	.51	.64	.63	.60	.66
	Disparity	.97	.91	1.02	1.21	1.06	1.38	1.04	.99	1.09
	Change in disparity	1.07	.99	1.15	.91	.79	1.06	.92	.86	.99

Note: All models adjusted for age, sex/gender, self-reported health (excellent, good, fair, or poor), and chronic conditions (asthma, arthritis, high blood pressure, diabetes, heart disease, cancer, stroke effects, anxiety disorders, and/or mood disorders.

That a slightly higher percentage of people with low income had consulted a medical professional compared to those with higher income (77.18% vs. 76.45%) in 2007/8 likely reflects greater need for care. In adjusted models, odds of consultation are lower for people with low household income. At the same time, the percentage of people with a regular medical provider or place of care was consistently lower among those with low income. Multivariable models reinforce that observed disparities in access are inequitable in that they do not align with need for care.

While access to primary care differs by sex/gender and province/territory, stratified analyses show that observed disparities and changes over time were consistent within sex/gender categories and provinces/territories. For this reason, we focus on national analysis.

## Interpretation

This analysis of national health survey data shows that disparities in access to primary care exist and have persisted or increased over time. These findings are consistent with existing studies highlighting disparities in access to primary care,^21,22,38^ while extending them to show that disparities have not attenuated over time. Findings add to observations of declining service use and widening disparities by neighbourhood income observed in health system data^19^ showing similar patterns with individual-level measures of social position and a different data source. Findings are also consistent with analysis of specific reform efforts that show differential access to reforms by income.^19-22^

The period captured between observations in this study saw primary care reform across all provinces and territories.^1,12-14^ Reform efforts involved a range of strategies to improve access to primary care, but equity was not an explicit objective.^13,14^ It is often observed that health systems with stronger primary care systems produce more equitable outcomes, based on comparisons across jurisdictions.^1-4^ Our findings highlight that primary care reforms that do not explicitly integrate equity as an objective are unlikely to impact equity in access to primary care, or, by extension, equity in health outcomes more broadly.^39-41^

There has been important work to outline dimensions of equity-oriented healthcare, including trauma- and violence-informed care, contextually tailored care, and culturally safe care at clinic or organizational level,^39^ but this is not yet widely implemented. While there are models of team-based care with express commitments to meeting the needs of the communities they serve in an equity-informed manner, such as community health centres,^42^ new investment in such models was limited over the study period.^13,14^ Other research has highlighted how equity-mandated organizations must navigate competing discourses within health systems, with equity framed as an “add-on” to usual primary care, rather than an integral and fundamental aspect of care.^43^ To support delivery of equity-oriented care, accountability and performance frameworks must fully align with an equity mandate and patterns of funding and resource allocation must be tailored to needs and responsive as needs change.^44^

Box 1. Summary of recommendations to support equity in access to primary care

• Include equity as an integral objective in primary care reform.
• Expand training and capacity for trauma- and violence-informed care, contextually tailored care, and culturally safe care.
• Support models of team-based care with equity mandates and accountability to communities, such as community health centres.
• Ensure equity mandates are captured in accountability and performance frameworks.
• Ensure patterns of funding and resource allocation are tailored to needs and responsive as needs change.

## Limitations

This analysis is limited in several ways. Measures available in the CCHS are limited. Access to a medical professional is not specific to primary care, question wording varied slightly in the early and later years, and there is no information about quality of care received. We can only observe if people are racialized as non-White or White and there is no measure of gender. This centres Whiteness and reinforces cisnormativity. Location of residence assigned at the level of health region likely obscures substantial differences in access within regions, including between urban, small town, rural and remote places. Measures of need are also limited, especially given that people must have received healthcare to have identified chronic conditions. This likely means we likely under-adjust for need in multivariable analyses. This research reveals only broad patterns and trends. Different information and close engagement with equity deserving communities is needed to understand access to care in greater depth and to plan more equitable primary care delivery.

CCHS data are particularly limited in their ability to inform primary care among Indigenous Peoples in Canada. Though inequitable access to preventative and primary care among First Nations, Inuit, and Métis people is documented elsewhere,^45^ Indigenous Peoples are invisible in this analysis. The CCHS does not include people living on reserves and only identifies Indigenous people following the 2015/16 survey. While there are innovative primary care reforms led by Indigenous communities and organizations that warrant study^44,46,47^ our analysis cannot capture impacts of innovations on health equity within communities and nations. Different approaches are needed to explore these.

We chose to make comparisons prior to the COVID-19 pandemic, but widespread changes to primary care, including rapid expansion of virtual options may have equity impacts that require careful study. Since the study period, it has become even more difficult to obtain consistent and reliable access to a primary care provider^48^ and provinces are considering various directions for future reform, so ongoing tracking of disparities is important.

## Conclusions

Primary care is receiving renewed attention, as multiple jurisdictions are struggling to make sure people can access needed care. This analysis suggests that approaches to primary care transformation that do not explicitly consider equity may continue to entrench inequities. Health leaders are encouraged to design primary care transformation efforts with equity as priority area of focus. This would require allocation of new investments in primary care to meet the needs of underserved populations and inclusion of equity in accountability frameworks. Careful study of the equity impacts of ongoing policy reforms is needed.
